# Revolutionizing Robotic Depalletizing: AI-Enhanced Parcel Detecting with Adaptive 3D Machine Vision and RGB-D Imaging for Automated Unloading

**DOI:** 10.3390/s24051473

**Published:** 2024-02-24

**Authors:** Seongje Kim, Van-Doi Truong, Kwang-Hee Lee, Jonghun Yoon

**Affiliations:** 1 Department of Mechanical Design Engineering, Hanyang University, 222 Wangsimni-ro, Seongdong-gu, Seoul 04763, Republic of Korea; chack11200529@gmail.com (S.K.); vandoitruong1997@gmail.com (V.-D.T.); 2 BK21 FOUR ERICA-ACE Center, Hanyang University, 55 Hanyangdaehak-ro, Sangnok-gu, Ansan-si 15588, Republic of Korea; 3 Korea Institute of Industrial Technology (KITECH), Ansan-si 15588, Republic of Korea; leekh@kitech.re.kr; 4 Department of Mechanical Engineering, Hanyang University, 55, Hanyangdaehak-ro, Sangnok-gu, Ansan-si 15588, Republic of Korea; 5 AIDICOME Inc., 55, Hanyangdaehak-ro, Sangnok-gu, Ansan-si 15588, Republic of Korea

**Keywords:** parcel detection, unloading system, RGB-D, machine vision, object detection, image segmentation

## Abstract

Detecting parcels accurately and efficiently has always been a challenging task when unloading from trucks onto conveyor belts because of the diverse and complex ways in which parcels are stacked. Conventional methods struggle to quickly and accurately classify the various shapes and surface patterns of unordered parcels. In this paper, we propose a parcel-picking surface detection method based on deep learning and image processing for the efficient unloading of diverse and unordered parcels. Our goal is to develop a systematic image processing algorithm that emphasises the boundaries of parcels regardless of their shape, pattern, or layout. The core of the algorithm is the utilisation of RGB-D technology for detecting the primary boundary lines regardless of obstacles such as adhesive labels, tapes, or parcel surface patterns. For cases where detecting the boundary lines is difficult owing to narrow gaps between parcels, we propose using deep learning-based boundary line detection through the You Only Look at Coefficients (YOLACT) model. Using image segmentation techniques, the algorithm efficiently predicts boundary lines, enabling the accurate detection of irregularly sized parcels with complex surface patterns. Furthermore, even for rotated parcels, we can extract their edges through complex mathematical operations using the depth values of the specified position, enabling the detection of the wider surfaces of the rotated parcels. Finally, we validate the accuracy and real-time performance of our proposed method through various case studies, achieving mAP (50) values of 93.8% and 90.8% for randomly sized and rotationally covered boxes with diverse colours and patterns, respectively.

## 1. Introduction

**Figure 1:**
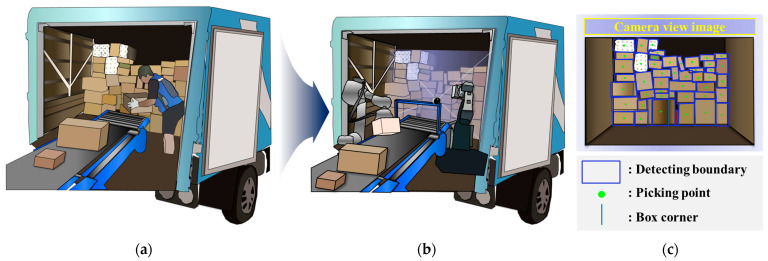
Conceptual diagram of parcel depalletisation: (**a**) conventional conveyor belt system based on manual labour, (**b**) proposed system supported by robot arms and vision sensors, and (**c**) image detected using 3D RGB-D camera for automatic picking.

With technical progress and tremendous increases in labouring cost, there has been extensive research work toward automation for the transportation and logistics industries. This trend has been accelerated by increasing demands in online shopping along with a pandemic such as COVID-19, which tend to require higher efficient logistics and delivering systems. Figure 1 demonstrates the procedure of courier delivery, encompassing the transportation of products from the truck to the product handling facility. This process is involved in the classification of items received by the courier at a national scale, subsequent delivery to the product handling facility, and final distribution to their respective destinations. The current system entails significant time and labour costs due to the manual handling of numerous and diverse packages directly from the truck to the conveyor belt. One way to address this burden is to develop a core technology that enables fast and accurate delivery through automated depalletizing [1,2,3,4] without the use of personnel. To achieve this, it is necessary to employ image processing methods [5,6,7] and deep learning techniques [8,9,10] that accurately and rapidly detect the locations and sizes of parcels. The current parcel detection method developed using the above two methods is based on learned data and is limited in terms of detection of size, layout, individual parcels, and patterns. It also has poor individual parcel detection abilities for overlapping arrangements. Therefore, this paper proposes detection techniques for various layouts and types, such as detection and segmentation of unlearned objects (Zhang et al. [11]). Another similar approach is the bin picking technique (Park et al. [12]), which involves the retrieval of desired objects from a pile of various items with different sizes and shapes. However, bin picking has the disadvantage of only recognising objects that have been previously trained, making it challenging to detect untrained objects. Using this technique, the system should be able to handle the detection of stacked parcels, rotated parcels, plain brown parcels, and parcels with coated patterns. Ultimately, it needs to process the detection of all parcels on the front of the truck within one second, including all the detections.

Early parcel detection studies used traditional image processing methods [13,14,15,16] to detect individual parcels using RGB or depth images. Yunardi et al. [15] placed two cameras on the upper and flat parts on the conveyor belt to detect the size of a parcel, captured a picture of the parcel on the conveyor belt, extracted the colour of the parcel as an RGB image, and detected the parcel through contour detection. The above method is possible in real time and has a detection rate of 87.5%, but it cannot detect overlapping or patterned parcels. Ladplee et al. [16] used a lidar camera, L515, mounted on the top of the stage to simultaneously capture multiple parcels with RGB-D images, then used the flood fill method to detect the parcels’ edges, positions, and sizes. This method has a parcel volume error rate of no more than 5% and a detection time of not more than 1 s. However, it is difficult for the above method to detect parcels at intervals of 20 mm or less; that is, image processing is very effective in detecting a set of objects in a formal environment, but it is difficult to detect amorphous or overlapping objects. Recent detection methods that have overcome these shortcomings include individual detection methods that use deep learning for object detection. Deep learning builds on convolutional neural networks (CNNs) to create candidate regions for objects to be detected in the original image, then identifies and tracks objects for each candidate region. The main object detection algorithms used are YOLO (Redmon et al. [17]) and R-CNN (He et al. [18]). YOLO is a grid-based approach that predicts boundaries and selects the highest-class probability after an object passes through a CNN, and R-CNN classifies the pixels that constitute the object in the identified boundaries, proposes the potential position of the object using a neural network, and classifies and detects the object based on the proposed area [19,20,21,22,23,24,25,26,27,28,29,30]. In the case of Zhao et al. [19], they propose a planar parcel detection method using R-CNN reinforcement. This method solves the problem of false detection by adding object edge loss to the loss function of the Fast R-CNN and improves detection accuracy by calculating feature pixel values using bilinear interpolation. The above method achieves a speed of 0.5 FPS and an accuracy of 98.76%. Han et al. [26] proposed a lightweight object detection network model for detecting randomly stacked parcels. This method detects randomly stacked parcels using the RGB-D and YOLOv3 models and estimates the optimal picking position of randomly stacked parcels using lightweight YOLOv3 models. At a detection speed of 50 FPS, the picking accuracy is 89.7%. Nguyen et al. [27] proposed a method to detect patterned parcels by standardising the parcel surface pattern using CycleGAN and creating boundary lines through image processing with depth, followed by detection using Mask R-CNN. However, this method shows a significantly lower detection accuracy when the gap between parcels is less than 15 mm, whereas it exhibits over 90% detection accuracy when the gap is 15 mm or more. The outer covers of real parcels have various shapes and patterns. As a result, it is difficult to detect new, unlearned parcels using the previous methods. None of these studies investigated how instance-level segmentation can detect the boundaries of a parcel to detect a courier. It is important to find a formalised area for courier detection with coverage of various shapes and patterns because the boundaries between parcels are standardised compared with the new parcel pattern.

In this paper, we propose a fast and novel deep learning-based method for image processing to detect the arrangement of multiple complex patterned coverings and unaligned parcels. First, we propose RGB-D-based image processing and parcel boundary detection using YOLACT’s image segmentation method (Bolya et al. [31]) to increase the detection accuracy of unaligned parcels with complex patterns. Second, we propose an image segmentation accuracy improvement with reference to the SAHI method (Hua et al. [32]) for the detection of unlearned parcels. Third, the detected parcel boundary is used to detect the parcels. Fourth, we propose an estimation method based on depth to detect the gripping position of the rotated courier. Finally, we propose a parallel algorithm of image processing and deep learning to improve the detection speed of the courier of a 5-ton truck. In a previous study by Yoon et al. [28], the depth map included segment boundaries and utilized CycleGAN to standardize the appearance of boxes, enabling box recognition through Mask-RCNN. In contrast, our proposed method focuses on box recognition through boundary segmentation rather than direct box detection using YOLACT. Consequently, despite using the Realsense L515 camera at a distance of 2000 mm, which outperforms the previous study’s ZIVID camera at a distance of 300 mm, our approach achieves a recognition rate of approximately 93% for boxes separated by a distance of 2 mm, compared with the previous study’s recognition rate of only around 20%. The remainder of this paper is organised as follows. It presents a framework of experimental conditions for implementing parcel classification methods, detailed methods of algorithms, overall experimental results and conclusions, and future work.

## 2. Experimental Conditions

### 2.1. Experimental Setup for Object Detection

**Figure 2:**
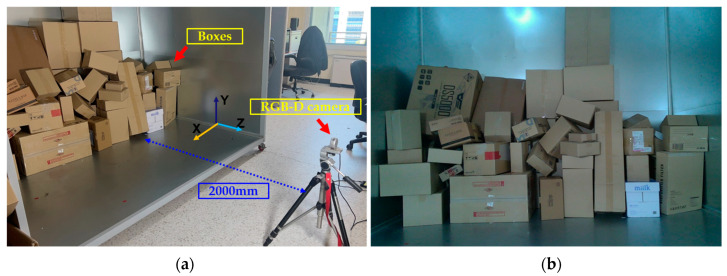
Experimental setup for parcel detection with RGB-D camera: (**a**) test condition with parcels and RGB-D camera and (**b**) observed RGB image.

During the training and testing processes of parcel and rotated parcel detection, we used a computer with an RTX 4080 GPU with 16 GB of video memory, 64 GB of memory, and an i9-13900K 24-core 32-thread CPU. The framework used for deep learning was Pytorch 1.13.1+cu116, CUDA 11.6, and most of the image processing framework used the OpenCV [33] library. All programs were implemented in Python 3.7. We set up an RGB-D vision-based robot sorting experimental platform in the laboratory to simulate actual express parcel sorting scenes and to confirm the accuracy and validity of the algorithm. The platform used a demo stage similar in size to a 5-ton truck (2300 mm × 2000 mm × 1800 mm) as the target express truck. The resolution of the two images, with an RGB resolution of 1280 × 720 pixels and a depth resolution of 1024 × 768 pixels, was calibrated to 1280 × 720 pixels at 30 FPS. We used a LiDAR camera (L515) with a depth range of 1800–2500 mm, considering the distance between the camera and the parcel as 2000 mm. To achieve precise parcel sorting, we used a 30 W spotlight. As shown in Figure 2 the sorting targets were parcels of different sizes and shapes randomly placed within the field of view of the RGB-D camera shown in Table 1.

**Figure 3:**
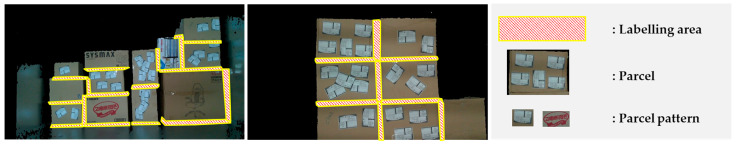
Test image for boundary detection in the machine learning process.

To train the object detection model, we learned the boundaries between parcels of various arrangements and colours through experiments. The experimental data were obtained using an RGB-D camera. A total of 2050 images were used for learning, and 50 images were used for verification. Finally, we manually labelled the parcel boundaries using Labelme (Torralba et al. [34]), which can label a specific area for object detection training. To increase the accuracy of the image, we did not label the area where the resolution of the image was poor, nor did we label the area between the parcels or any part other than the parcel and the wall shown in Figure 3.

### 2.2. Verification Method for Parcel Detection

Our method aims to improve the detection performance of densely stacked parcels, regardless of the pattern and rotation of the parcels. The detection method utilises YOLACT, which segments the trained object; hue, saturation, and value (HSV); and depth images to determine the boundaries of a parcel and detect individual parcels. To validate the detection performance, we followed the method in Sokolova et al. [35] and used precision (*Pr*), recall (*Re*), average precision (*AP*), and processing time as metrics. True positives (*TP*), false negatives (*FN*), and false positives (*FP*) were calculated to generate the recall and precision. The precision Equation (2) defines *TP* as the percentage of all predictions that are positive, while the recall Equation (1) is a metric that measures how well the model detects all samples that are actually positive.

(1) $$Re=\frac{TP}{TP+FN}$$

(2) $$Pr=\frac{TP}{TP+FP}$$

The *AP* Equation (3) is a metric that represents the performance of object detection algorithms as a single value, calculated as the area under the precision–recall curve. In computer vision, the performance of object detection algorithms is typically evaluated using *AP*.

(3) $$AP=\int_{0}^{1}PRdr$$

The above evaluation method is expressed as an intersection over union (IOU) threshold. IOU is a metric that determines whether the detection of an individual object is successful and has a value between 0 and 1. This method is shown in Rezatofighi et al. [36].

We measured the parcel detection rate for each configuration using 50 images. The sizes of the parcels ranged from a minimum of 500 mm (width + height + depth) to a maximum of 1600 mm (width + height + depth), and each side of the parcels must be within 1000 mm.

### 2.3. Parcel Arrangement Environment for Actual Experiments

To verify the basic parcel detection accuracy of image processing and YOLACT, we used a vertical parcels dataset with the camera. Using this method, we could determine how well each parcel was divided and detected.

**Figure 4:**
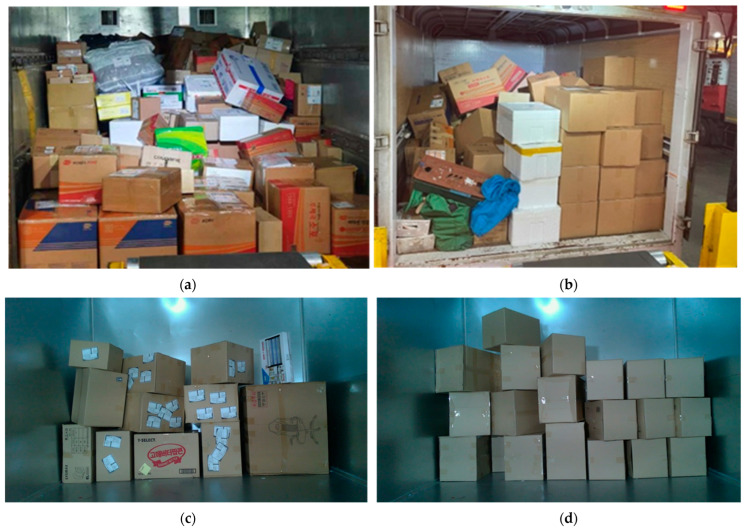
Comparison of depalletisation environments: (**a**) in actual practice with a complex pattern, (**b**) in actual practice with a simple pattern, (**c**) in demonstration site with a complex pattern, and (**d**) in demonstration site with a simple pattern.

To validate the robustness of our method, we used a dataset that simulated conditions similar to those in actual parcel unloading. The parcels were constructed with random numbers, sizes, patterns, and arrangements. For rotated parcels, we checked the detection of the wider face among multiple faces shown in Figure 4.

## 3. Detection Method for Complex Array of Parcels in Delivery Truck

**Figure 5:**
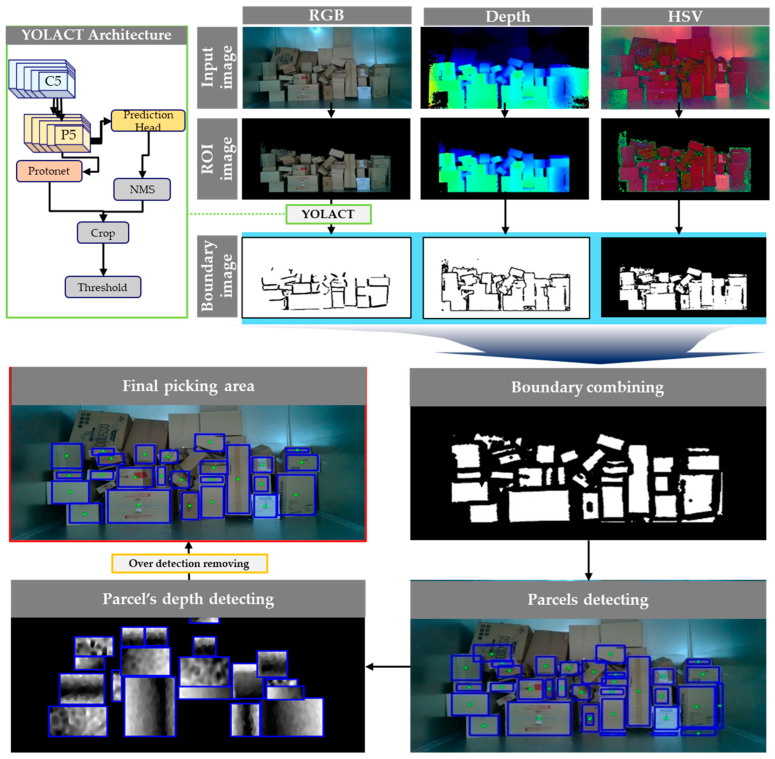
Overall flow chart based on deep learning and image processing in depalletising process.

The overall framework of the parcel-sorting method based on deep learning and image processing is shown in Figure 5 The background of the stacked parcels was removed using the specified depth map area. Then, a specific area was extracted from the HSV image for boundary detection, and the boundaries were detected in the depth and RGB images using YOLACT. The integrated edges were used to detect the position of the parcels, and the corners of the rotated box were extracted from the depth image to identify the centre point of the wider area of the detected parcels. In this case, the robot’s grip range was 2000 mm, so it first recognised the front box, which was inside 2300 mm, taking into account the rotated box. After all the front boxes were recognised and grasped, if no objects were recognised through the depth map, the robot and camera entered the truck together to recognise the parcels in the back.

**Figure 6:**
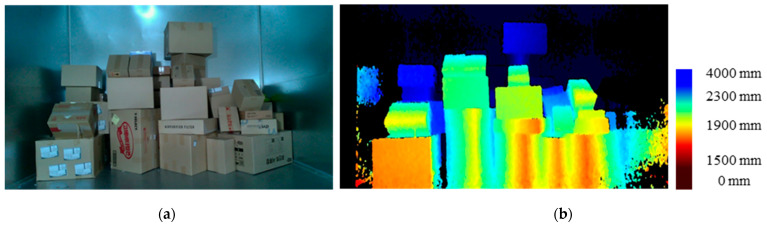
Physical test image with parcels piled up: (**a**) RGB image and (**b**) depth map from RGB-D camera.

The first step in object detection was to remove all noise while preserving the object, where noise was background from non-parcel delivery trucks, objects behind the robot’s grip limit, etc. To remove noise, only objects within a given distance were represented through depth. For parcel detection, we set the depth detection distance between 500 mm and 2300 mm to detect the frontmost parcel. The RGB and depth images captured by the camera are shown in Figure 6.

**Figure 7:**
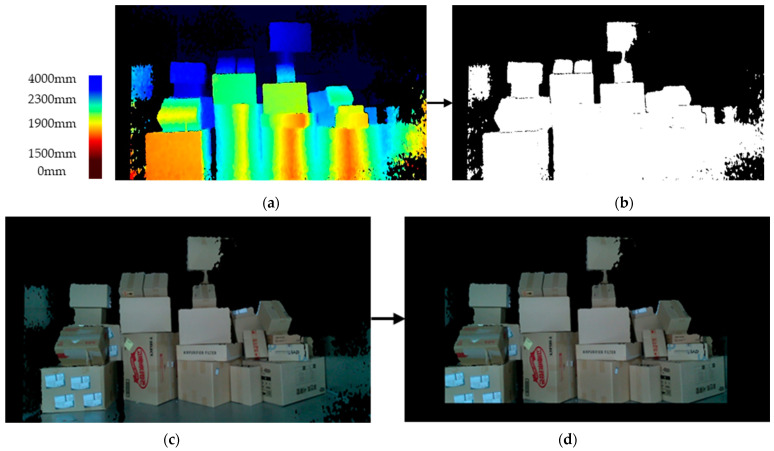
Background removal process for boundary highlighting: (**a**) depth image map, (**b**) binarisation of depth map, (**c**) enhanced image with elimination of background noise, and (**d**) final processed image focused on ROI.

A mask was created by binarising the detected depth image to remove parcels and backgrounds outside the designated distance using THRESH_BINARY (Equation (4)). When the generated mask was applied to the RGB image, parts of the parcels and the background were removed. Additionally, to eliminate areas other than parcels from the parcel truck, the distance between the camera and the parcel truck was calculated to remove a certain area and focus on the region of interest (ROI) shown in Figure 7.

(4) $$dst(x,\;y)=\left\{\begin{array}{c} maxval\;if\;src\left(x,y\right)>thresh\;\\ 0\;otherwise \end{array}\right.$$

### 3.1. Three Parcel Boundary Detection Methods for Boundary Integration

Parcels were detected using a boundary identification method, which was capable of detecting parcels with varying colours and patterns that had not been pretrained. The depth boundary detection method was effective for boundary extraction when the distance between parcels was greater than 10 mm. Conversely, the HSV and YOLACT boundary detection method was effective when the distance between parcels was less than 10 mm. By combining these detection methods, the entire boundary could be extracted with confidence.

**Figure 8:**
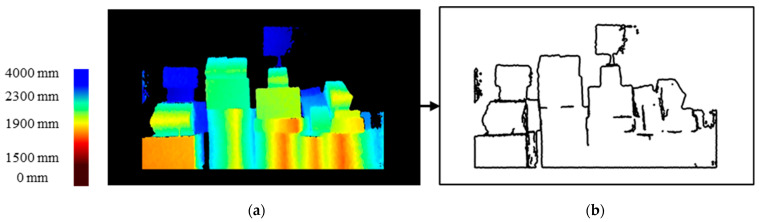
Boundary detection process using depth: (**a**) depth map with background removed and (**b**) Canny detection on depth map.

When there was a gap between two parcels, the depth map showed a difference, and when the distance between the parcels was greater than 50 mm, detection was possible. The Canny edge detection method was used to detect the boundary of the gap between parcels. However, the thin white boundary lines that were extracted made it difficult to properly divide the boxes; therefore, the dilation Equation (6) was used to increase the thickness of the boundary lines. For the expansion equation, we used MORPH_CROSS (Equation (5)) and a kernel with a size of (3,3). Then, to use the portion outside the boundary lines as a mask, the image with the detected boundary lines was inverted to change the white boundary lines to black and the black portion to white shown in Figure 8.

(5) $$E_{ij}=\left\{\begin{array}{c} 1\;if\;i=anchor.y\;or\;j=anchor\;.x\\ 0\;otherwise \end{array}\right.$$

(6) $$Dilate\;(x,y)\;=\;\underset{(i,j)\in kernel}{max}src(x+i,y+j)$$

**Figure 9:**
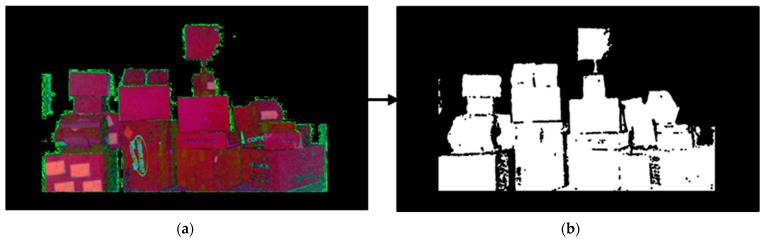
Boundary detection process based on HSV image: (**a**) HSV image with background removed and (**b**) HSV image colour extraction.

If a parcel was wrapped, it created a shadow. An HSV image was used to extract the shadow colour. Because the colour of the shadow may vary depending on the parcel’s colour, colours in the range (0,0,0) < HSV < (60,60,60) were extracted and represented in black. Additionally, if the extracted boundary line did not correctly represent the line, the erode Equation (7) was used to increase the size of the black areas to connect them to each other and create lines shown is Figure 9.

(7) $$Erode\;(x,y)\;=\;\underset{(i,j)\in \mathrm{kernel}}{min}src(x+i,y+j)$$

**Figure 10:**
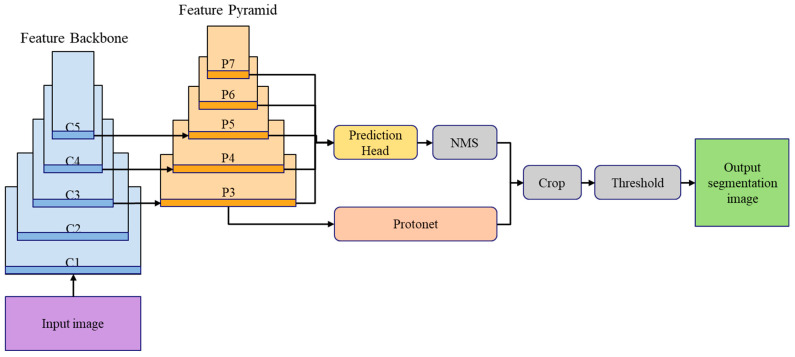
YOLACT architecture for boundary detection.

To detect the gap between parcels, we used YOLACT, a deep learning method that performed object segmentation to generate masks corresponding to the size of the detected object shown in Figure 10, in contrast to traditional object detection methods, which typically use bounding boxes to localise objects.

**Figure 11:**
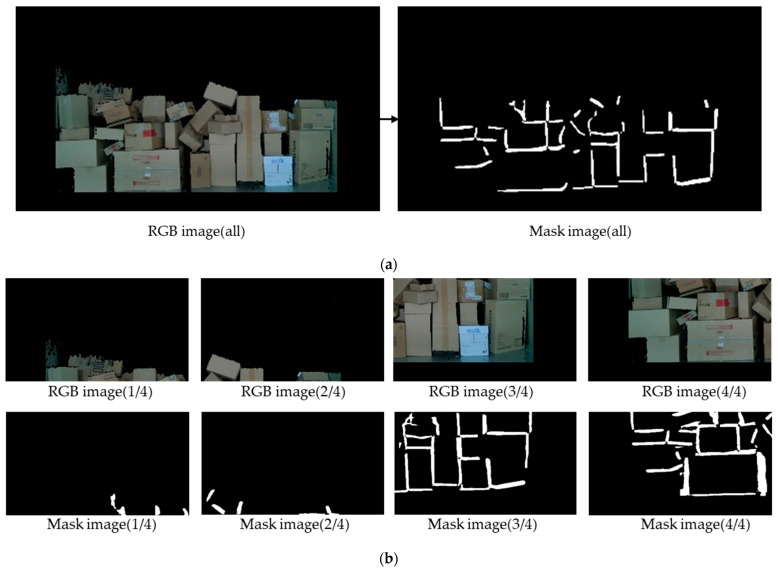
Boundary detection through YOLACT: (**a**) full image and (**b**) after cropping the image.

The learned boundary dataset was utilised to extract the boundaries of the pre-cropped images. However, owing to the large and diverse number of parcels in the images, it was difficult to detect all boundary lines. To address this issue, the image was divided into four parts, and boundary lines were detected again, as shown in Figure 11b, using the SAHI (Hua et al. [32]) method. This resulted in the extraction of a greater number of boundary lines. This process took approximately 0.18 s per image, but to perform real-time parcel detection, at least one image must be detected per second. Therefore, each image was divided into four parts, resulting in the detection of five images in total.

### 3.2. Parcel Detection for Boundary Contour Detection

**Figure 12:**
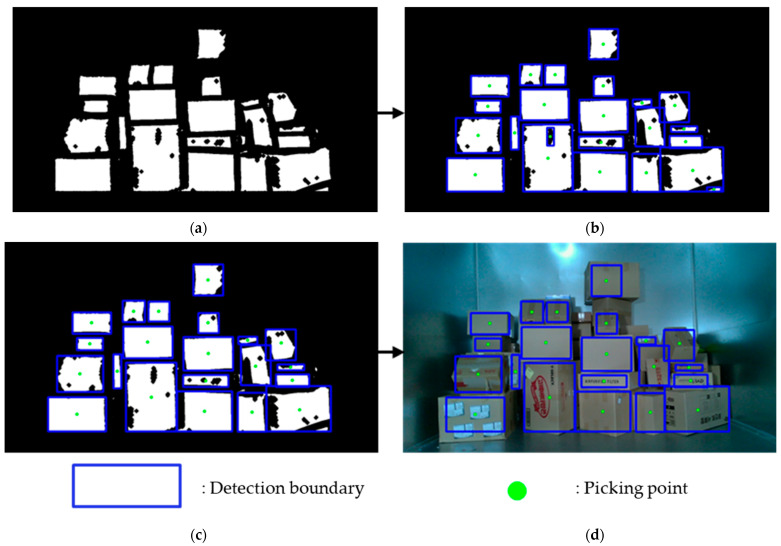
Parcel location and size detection: (**a**) synthesised boundary masks from YOLACT, HSV, and depth boundary segmentation; (**b**) contour detection of the boundary segmentation masks; (**c**) deletion of internal redundant contours; and (**d**) parcel detection by detected contours.

The process of integrating all detected boundary masks involved placing the largest HSV boundary mask as the background and overlaying the YOLACT boundary mask onto the HSV boundary mask area. Subsequently, the depth boundary mask was added to the mask area. The resulting image with all boundary masks integrated is shown in Figure 12a. Contours within the range of 100–2000 pixels were detected from the integrated boundary image, and by using the detected contours, a blue rectangle could be drawn to predict the shape of the parcel. Additionally, the centre point of the rectangle could be used to predict the picking points of the parcels. However, in this case, as shown in Figure 12b, additional boxes may be detected within the detected box due to excessive boundaries. To address this issue, we applied a method to remove noise within the box by comparing the four corners of the detected rectangles. If all four corners of a rectangle were located within the area of another rectangle, the detection was deleted.

### 3.3. Segmentation of the Wide Side of the Detected Parcel Using Depth

**Figure 13:**
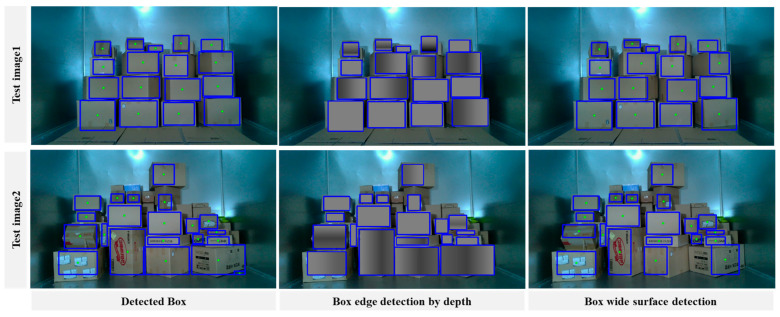
Box edge detection algorithm for wide surface remaining.

The degree of rotation of the predicted parcel box was indicated by a legend based on the measured depth, using the shape of the parcel box as a reference. Each predicted image of the bounding box was binarized to mark only the black part to detect the edge of the rotated area. The edges of the depth were then determined, and the detected contour rectangle was divided into smaller segments with lines. Among the divided contours, the larger contour was detected as the wider side of the rotated parcel box, and the centre point was extracted to predict the wider side and the picking point. If the contours had the same area with their edges facing the camera, an arbitrary adjustment was performed to recognise the left side. A conceptual diagram is shown in Figure 13.

### 3.4. Effective Noise Removing for Suppressing Over-Detection Using Area Calculation

**Figure 14:**
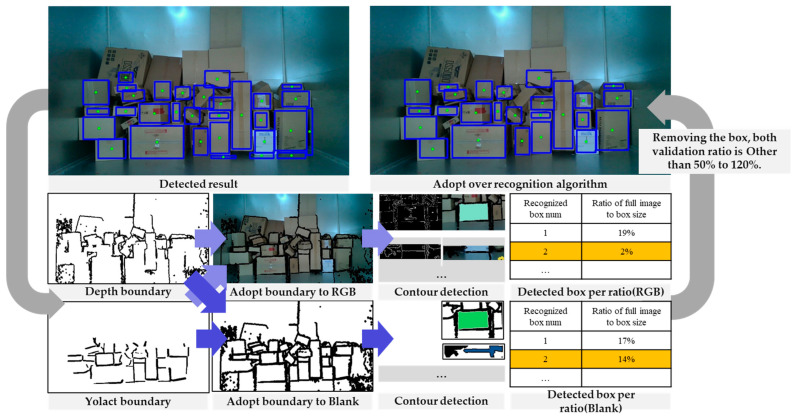
Flow chart of over-detection removal based on comparing contour area.

Because of the characteristic of box recognition through boundaries, it is possible to detect a greater number of boxes than the ground truth. This outcome significantly affects the overall box detection rate and can lead to issues during the actual unloading of packages. Therefore, it is crucial to address this issue. To resolve this, we verified the bounding boxes and eliminated predicted regions that were not actual boxes. Noise was most commonly observed at the edges of the recognised regions, mainly due to ROI extraction. To mitigate this, we overlaid the boundary surfaces obtained through depth images and YOLACT onto the RGB images with a white background, excluding the ROI. The overlaid image was then resized to 0.6 times the width and height ratio compared with the region where boxes were detected. For the cropped image, we first applied a blurring effect due to the pattern of the boxes and then detected the boundaries. In cases where the boundaries were correctly detected for a box, the area and length of the boundaries should be similar to those of the previously recognised box. Therefore, the boundary area should fall between 50% and 120% of the area of the previously recognised box. However, in some cases, despite the blurring effect, the correct box area could be below 50% due to the box pattern. Therefore, we employed a second approach, where we applied the same procedure to the white background containing the boundary surfaces from the depth and YOLACT. If the boundaries of the detected boxes were below 50% and over 120% in both the RGB and white background images, they were considered as noise and subsequently removed. Furthermore, in cases where the box was rotated along the X or Y axis, there were instances where the boundaries of the box were not detected. To address this issue and distinguish between actual boxes and noise, any rotated boxes with a detected boundary image width and height of at least 15 pixels were designated to be retained and not removed. We illustrate this algorithm in Figure 14.

## 4. Mean Average Precision Validation for Picking Point

The application of the depth algorithm for detecting a rotated parcel enabled the accurate identification of the wider side of the parcel. For comparison, without the depth algorithm, the entire parcel was represented as a rectangular box. However, with the application of the depth algorithm, the boundary line and centre point of the wider side of the parcel could be detected based on the corners of the parcel relative to the camera.

**Figure 15:**
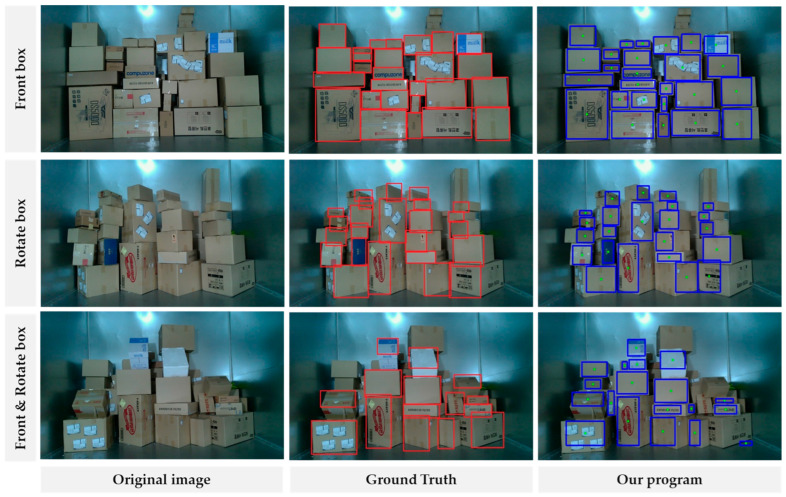
Demonstration results for separate parcels with respect to size and surface pattern.

To validate the model, we conducted experiments using three different setups in the laboratory. The first setup involved positioning the boxes at a frontal angle with respect to the camera. In the second setup, the boxes were randomly rotated along the X, Y, and Z axes. The third setup consisted of a combination of half the boxes positioned at a frontal angle and the other half randomly rotated. Each setup comprised 50, 25, and 50 images, for a total of 1043, 584, and 1084 parcels, respectively. Furthermore, for boxes placed at the camera’s frontal angle, the rotation angle was set to 0 degrees. In contrast, randomly rotated boxes were subjected to angles ranging from 30 degrees to 60 degrees. As a result, we successfully detected 1006, 562, and 1050 boxes in each setup, with an IOU of 50% or higher. The corresponding mean average precision (mAP) values for IOU values over 50% were 93.82%, 87.2%, and 90.85%. The results can be found in Figure 15 and Table 2.

Our detection method achieved high accuracy and a high IOU but operated at 1.42 FPS, meaning that it could process one image per second. However, this speed was deemed satisfactory for picking up parcel boxes.

## 5. Discussion

For comparison with the related literature on box detection, we conducted experiments in a similar environment to that of Zhao et al. [19], which focuses on the detection of non-rotated frontal boxes with various patterns. In their study, they achieved a mAP of 98.42%, while our program achieved 93.82%, showing a difference of approximately 4.5%. However, in comparison with the literature related to rotated box detection, such as S. Han et al. [26], where random boxes without specific rotated patterns were placed, our program demonstrated significantly improved accuracy with a recognition rate of 96.55% compared with their 87.9%. Moreover, our mAP reached 94.48%. Through these comparisons and the overall results, our program demonstrated the capability of detecting rotated boxes, making it applicable to real-world scenarios where packages may be disarranged due to vibrations during transportation on delivery trucks. Future research directions will involve developing the system for practical parcel depalletisation, designing new grippers to handle parcels of varying shapes, and integrating them into the current system to ensure successful unloading. For that reason, we will select the priority gripping point when we send the picking points to the robot system.

## 6. Conclusions

Parcel detection is a crucial component of AI-based automated robot unloading. Parcels in logistics centres are arranged in various coverings and complex and rotated arrays within delivery trucks, making individual detection difficult. To address this issue, we propose a method for detecting parcels by detecting their boundary surfaces using image processing and AI. Our main contributions are as follows.

(1) First, we detected parcel positions by detecting boundary surfaces instead of using general object detection; this process detected parcels using deep learning-based boundary detection and box detection for various coverings and arrays. Boundary detection was performed by extracting boundary surfaces from depth maps using Canny edge detection, extracting specific colours from HSV images, and detecting boundary surfaces from RGB images using YOLACT. The locations of the parcels were then identified by combining all detected boundary surfaces and contours.

(2) Second, we applied a method for detecting the corners of the rotated parcels based on depth. By comparing the relative distances of sharp changes in depth within the detected individual parcels, we identified the corners. The picking point of the robot could be extracted by detecting the corners and boundary surfaces of the rotated parcels.

(3) Third, we conducted tests on a parcel dataset. Our method achieved mAP values of 93.82%, 87.2%, and 90.85% for each case and an FPS of 1.42 on the parcel dataset. The experiment showed that parcel detection was possible on an XY plane of 2300 mm × 1500 mm with a camera distance of 2000 mm.

## Figures and Tables

**Table 1:** Specifications of RGB-D camera.

Information	Model	Resolution (RGB)	Resolution (Depth)	FPS
Camera	Intel L515 (RGB-D)	1920 × 1080	1024 × 768	30

**Table 2:** Quantitative analysis of rotated parcel box detection.

Box Arrangement	Image Number	Parcel Number	Precision (%)	Recall (%)	mAP (0.5) %	FPS
Front box	50	1043	96.26	96.6	93.82	1.42
Rotated box	25	584	90.2	96.23	87.2	1.43
Front and rotated box	50	1082	92.3	95.2	90.85	1.41

## Data Availability

Data are available with the permission of all authors.
